# Gastrointestinal Dysfunction and Low‐Grade Inflammation Associate With Enteric Neuronal Amyloid‐β in a Model for Amyloid Pathology

**DOI:** 10.1111/nmo.15016

**Published:** 2025-03-06

**Authors:** Kinga Réka Tasnády, Reindert Jehoul, Manuel Gutiérrez de Ravé, Marion J. Gijbels, Bert Brône, Ilse Dewachter, Veerle Melotte, Werend Boesmans

**Affiliations:** ^1^ Biomedical Research Institute (BIOMED) Hasselt University Diepenbeek Belgium; ^2^ Department of Pathology, GROW‐Research Institute for Oncology and Reproduction Maastricht University Medical Centre Maastricht the Netherlands; ^3^ Department of Pathology, NUTRIM Institute of Nutrition and Translational Research in Metabolism Maastricht University Medical Centre Maastricht the Netherlands; ^4^ Department of Medical Biochemistry, Experimental Vascular Biology, Amsterdam Infection and Immunity, Amsterdam Cardiovascular Sciences Amsterdam University Medical Center Amsterdam the Netherlands; ^5^ Department of Clinical Genetics Erasmus University Medical Center Rotterdam the Netherlands

**Keywords:** enteric glia, enteric nervous system, gut function, intestinal motility, neuroinflammation

## Abstract

**Background:**

Patients suffering from Alzheimer's disease, a progressive neurodegenerative disorder involving cognitive decline and memory impairment, often present with gastrointestinal comorbidities. Accumulating data also indicate that alterations in the gut can modulate Alzheimer's disease pathology, highlighting the need to better understand the link between gastrointestinal abnormalities and neurodegeneration in the brain.

**Methods:**

To disentangle the pathophysiology of gastrointestinal dysfunction in Alzheimer's disease, we conducted a detailed pathological characterization of the gastrointestinal tract of 5xFAD mice by performing histological analyses, gene expression studies, immunofluorescence labeling and gut function assays.

**Results:**

We found that 5xFAD mice have elevated levels of intestinal amyloid precursor protein and accumulate amyloid‐β in enteric neurons. Histopathology revealed that this is associated with mild intestinal inflammation and fibrosis and accompanied by increased expression of proinflammatory cytokines. While overall enteric nervous system composition and organization appeared unaffected, 5xFAD mice have faster gastrointestinal transit.

**Conclusion:**

Our findings indicate that amyloid‐β accumulation in enteric neurons is associated with low‐grade intestinal inflammation and altered motility and suggest that peripheral pathology may cause gastrointestinal dysfunction in Alzheimer's disease patients.

## Graphical abstract

Amyloid‐β accumulation in enteric neurons is associated with low‐grade intestinal inflammation, fibrosis, and altered motility in a mouse model for amyloid pathology, suggesting that peripheral pathology may cause gastrointestinal dysfunction in Alzheimer's disease patients.

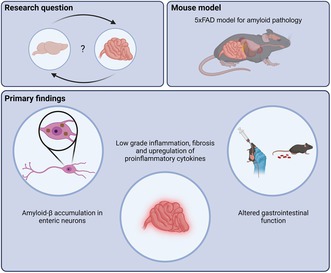

**Summary**

- Growing evidence demonstrates the involvement of the gut‐brain axis in Alzheimer's disease (AD). To investigate the role of peripheral pathological changes in AD, we utilized the 5xFAD mouse model, exhibiting neuronal overexpression of mutant human amyloid precursor protein‐695 and presenilin‐1 transgenes.
- In 5xFAD mice, increased intestinal amyloid precursor protein expression and amyloid‐β accumulation in enteric neurons were observed, alongside mild intestinal inflammation and fibrosis.
- Despite these pathological changes and accelerated gastrointestinal transit time, the composition and organization of the enteric nervous system remained unaffected in 5xFAD mice.

## 1 Introduction

Alzheimer's disease (AD) is a progressive neurodegenerative disorder primarily affecting the central nervous system (CNS), entailing cognitive decline and memory impairment [1]. AD is categorized into familial or early‐onset AD, which is associated with genetic factors, and late‐onset AD, where environmental influences and lifestyle are believed to be crucial [2, 3]. The mutations primarily associated with early‐onset AD occur in amyloid precursor protein (*APP*), presenilin 1 (*PSEN1*), and presenilin 2 (*PSEN2*) [4]. Brains of AD patients are characterized by the presence of amyloid plaques and neurofibrillary tangles, composed of amyloid‐β (Aβ) peptides and hyperphosphorylated Tau protein, respectively [5, 6]. Although the pathophysiology of AD remains to be elucidated, it involves chronic neuroinflammation, oxidative stress, and disrupted calcium homeostasis, contributing to synaptic dysfunction and neurodegeneration [7].

Neurodegenerative disorders, including AD, are often associated with gastrointestinal dysfunction [8] manifesting in symptoms such as constipation [9], delayed gastric emptying [10, 11], dysphagia [12], and malabsorption [13]. Whether these gastrointestinal abnormalities are caused by peripheral AD‐like pathology is not clear. Nevertheless, while APP is highly expressed in the CNS, it is also present in peripheral tissues, including the gut [14]. Moreover, Aβ accumulation has been detected in the intestine of AD patients [14, 15], suggesting a possible role for Aβ pathology in gastrointestinal dysfunction. Next to the generation of gastrointestinal comorbidities, alterations in the gut are also hypothesized to modulate CNS pathology and neurodegeneration [16]. Recent studies involving human cohorts demonstrated an association between the frequency of bowel movements, gut microbiota, and cognitive function [17], and found that constipation is associated with an increased risk for AD [18]. With an increasing number of reports pointing to a role for the microbiota‐gut‐brain axis [19], a better understanding of the reciprocal link between the gut and the brain in AD has become crucial.

To resolve the mechanisms underlying gastrointestinal symptom generation in AD patients, various AD mouse models have been employed [20]. Generally, these AD mouse models exhibit changes in gastrointestinal motility and intestinal permeability [18, 21, 22, 23, 24, 25, 26], mimicking observations in AD patients to various extents. Interestingly, using the 5xFAD mouse model, in which neurons overexpress mutant human *APP695* and *PSEN1* transgenes [27], Stoye et al. observed accelerated whole gut transit in 21‐ and 40‐week‐old mice [24]. Confirming the faster gastrointestinal transit time, Nguyen et al. uncovered that in 5xFAD mice, this is accompanied by a decrease in the thickness of the muscularis in the duodenum [25]. Notably, using the same model, others observed slower whole gut transit [18] or found no alteration in gastrointestinal motility ex vivo [26]. Various studies also report different findings on how the enteric nervous system (ENS) is affected in AD mouse models [28, 29, 30]. The characterization of the ENS of 5xFAD mice, in particular, is not yet fully elucidated. Observations range from diminished activity of acetylcholinesterase within the small intestine and increased expression of glial fibrillary acidic protein (GFAP) in the colon [24] to alterations in colonic ENS architecture [25]. Furthermore, while studies involving other AD mouse models have consistently observed activation of proinflammatory processes and immune cell infiltration in the gut [28, 31], intestinal inflammation in 5xFAD mice has not been reported. In addition, while other AD mouse models substantiate either the presence of Aβ within myenteric neurons [21, 28, 29], or report Aβ accumulation in the ENS [23, 31, 32], in the 5xFAD model, findings regarding Aβ are limited and contradictory [24, 25, 26]. This requires clarification since pathophysiological APP cleavage, which results in Aβ peptide, could render enteric neurons susceptible to damage, potentially leading to ENS dysfunction in the context of AD [31].

For these reasons, in this study, we aim to provide a detailed pathological characterization of the gastrointestinal tract of 5xFAD mice, with particular attention to the presence and location of Aβ accumulation in the gut and its relation to gastrointestinal function and ENS composition.

## 2 Materials and Methods

### 2.1 Animals

Female and male 6‐month‐old hemizygous 5xFAD mice, expressing human *APP* and *PSEN1* transgenes, driven by the thymocyte differentiation antigen 1 (*Thy1*) promoter, were used in comparison to their wild‐type littermates (WT mice). The 5xFAD mice, generated by the group of R. Vassar [27], harbor five AD‐linked mutations: the Swedish (K670N/M671L), Florida (I716V), and London (V717I) mutations in *APP*, and the M146L and L286V mutations in *PSEN1*. Animals were housed under regular conditions on a 12 h:12 h light–dark cycle in temperature‐controlled rooms (20°C ± 3°C) with access to autoclaved water and food *ad libitum*. All mice were maintained in filter‐top cages provided with cage enrichment items. Mice were sacrificed by cervical dislocation, unless mentioned otherwise. All experiments were performed in accordance with Belgian regulations and conducted with the approval of the Ethical Committee for Animal Welfare of Hasselt University.

### 2.2 Histochemistry

Hematoxylin and eosin (H&E) and Sirius red stainings were performed on 4 μm sections of formalin‐fixed paraffin‐embedded small intestinal and colon Swiss rolls. All images were acquired using a Leica DM3000 microscope equipped with a Leica DFC320 camera and examined for pathology.

### 2.3 Whole Gut Transit Time Assay

Total gastrointestinal transit time was measured as previously described [33]. Prior to the experiment, which was consistently performed in the late afternoon, each mouse was weighed and individually housed without bedding, with only access to water *ad libitum*. After 1 h of fasting, 0.3 mL of 6% (w/v) carmine red dye (Sigma‐Aldrich, ref. C1022) with 0.5% (w/v) methylcellulose (Sigma‐Aldrich, ref. M0512), diluted in sterile 1x phosphate‐buffered saline (1xPBS), was administered to each mouse by oral gavage. Subsequently, mice were placed back individually with access to water and chow. The time period from gavage until the emergence of the first red‐colored fecal pellet was recorded as the total gastrointestinal transit time. Following this, animals were returned to their home cage. If no pellet had been delivered 5 h post gavage, the experiment was discontinued.

### 2.4 Intestinal Permeability Assay

Mice were weighed and single‐housed with access to water *ad libitum* but not to food prior to the experiment. After 2 h, 4 kDa FITC‐Dextran solution, consisting of 0.6 mg FITC‐Dextran (Sigma‐Aldrich, ref. 46944) per gram body weight dissolved in sterile 1xPBS, was administered via oral gavage. 2 h post gavage, mice were anesthetized by intraperitoneal administration of a mixture of ketamine 10% w/v (Nimatek), xylazine 2% w/v (Rompun, Bayer), and sterile 1xPBS (1 and 0.12 mg/10 g body weight ketamine and xylazine, respectively, dose volume 0.1 mL/10 g). Thereafter, a cardiac puncture of the right ventricle was performed with a Heparin‐rinsed syringe (LEO), and a minimum of 500 μL blood was collected per animal. Blood samples were centrifuged for 10 min at 10,000× *g* at 4°C, and plasma samples were used for determining FITC‐Dextran content in 1:1 dilution with sterile 1xPBS. Fluorescence was determined using a CLARIOstar Plus (BMG Labtech) plate reader, using bottom detection, 485 nm excitation, and 528 nm emission wavelength.

### 2.5 Stool Analysis

During fasting steps of the in vivo assays, conducted while the mice were individually housed, stool samples were collected. For the Mouse lipocalin‐2 Enzyme‐Linked Immunosorbent Assay (ELISA), two fecal pellets per mouse were placed in sterile 500 μL 0.1% Tween 20 (Sigma‐Aldrich, P1379):1xPBS solution and stored at −80°C until further processing. For basic stool analysis, stools produced over a 1‐h period were collected, and the average total weight per stool was measured. The fecal samples were then allowed to desiccate at 75°C overnight to determine their dry weight. Water content per stool was calculated as the difference between wet and dry weight.

### 2.6 Lipocalin Measurements

After thawing the stool samples and centrifugation at 10,000× g for 10 min at 4°C, supernatants were collected and diluted 1:50 in sterile Reagent Diluent (R&D System, DY995), and measurements were carried out following the manufacturer's instructions (R&D Systems, DuoSet ELISA Development System Mouse Lipocalin‐2/NGAL, DY1857‐05). As the substrate solution and stop solution were excluded from the kit, the experiment was carried out by using these reagents from a Tecan kit (Corticosterone ELISA kit, RE52211). Optical density measurements were performed on a CLARIOstar Plus (BMG Labtech) plate reader at 450 nm, and the wavelength correction was measured at 540 nm.

### 2.7 Quantitative Real‐Time PCR

Whole gut samples (for tumor necrosis factor alpha (*Tnf‐α*), interleukin‐1 beta (*Il‐1β*), and interleukin‐6 (*Il‐6*) mRNA expression measurements) and myenteric plexus preparations (for *APP*, S100 calcium binding protein B (*S100β*), and *Gfap* mRNA expression measurements) were isolated and snap‐frozen in liquid nitrogen. Tissue samples were homogenized and lysed using QIAzol lysis reagent (Qiagen). Next, RNA was extracted using the RNeasy mini kit (Qiagen) according to the manufacturer's instructions. The quantity and quality of the RNA were measured using a NanoDrop2000 spectrophotometer (Isogen Life Science). Using qScript cDNA SuperMix (Quantabio), RNA was converted to cDNA according to the manufacturer's protocol. The reverse transcription reaction was performed with a T100 Thermal Cycler (Biorad). Using fast cycling conditions, quantitative real‐time PCR (RT‐qPCR) was carried out on a QuantStudio3 detection system (Applied Biosystems). By using the 2^−ΔΔCt^ method, relative quantification of gene expression was calculated and normalized using glyceraldehyde‐3‐phosphate dehydrogenase (*Gapdh*) and Phosphoglycaerate kinase 1 (*Pgk1*) as reference genes. Primer sequences are summarized in Table S1.

### 2.8 Immunofluorescence of Myenteric Plexus Preparations

To visualize ENS composition and Aβ accumulation in the myenteric plexus, intestinal samples were collected in ice‐cold 1xPBS, pinned down onto Sylgard‐coated petri dishes (Sylgard 184 Elastomer, Dow Corning) and fixed in 4% paraformaldehyde:1xPBS solution overnight at room temperature. After fixation and washing with ice‐cold 1xPBS, excess fat was removed, and an incision was made along the mesenteric border. The intestinal segments were then opened, and the mucosa, submucosa, and the circular (for both small intestine and colon) and longitudinal (for colon) muscle layers were removed using fine forceps. Small pieces (± 5 mm) of myenteric plexus preparations were permeabilized and blocked with 1% triton X‐100:1xPBS containing 4% goat or donkey serum (12.5:0.5 mL ratio) for 2 h at room temperature. For the Aβ markers, the immunodetection protocol started with an antigen retrieval step (HistoVT One, Nacalai Tesque), followed by 1xPBS washing and permeabilization using 0.1% Tween 20 (Sigma‐Aldrich, P1379) in 1xPBS for 30 min at room temperature. Subsequently, the tissue specimens were incubated on a shaker with adequate primary antibodies diluted in blocking medium overnight at 4°C. For WO2 and 4G8, this incubation step lasted for two nights. After primary antibody labeling, all preparations were washed in 1xPBS and incubated in blocking solution containing matched secondary antibodies at 4°C for 2 h. After washing the tissue samples, the preparations were mounted onto glass slides using Fluoromount‐G (Invitrogen). Laser scanning confocal microscopy (Figure 2) was performed on a Zeiss Axio Observer 7 inverted microscope equipped with an LSM 900 confocal scanhead, a Plan‐Apochromat 20×/NA 0.8 objective lens, and 561 and 640 nm diode lasers. Additional zoom and the number of pixels were balanced to fulfill the Nyquist criterion, resulting in a pixel scaling of 171 × 171 nm. Laser scanning was performed unidirectionally with a pixel dwell time of 4.52 μs (no averaging), and emission was detected using the internal GaAsP‐PMT detectors. Images were sequentially acquired using 640 and 561 nm excitation (~21 μW and ~14.5 μW before the objective lens, respectively), and acquisition channels were configured as follows. Alexa Fluor 647: 645–700 nm emission wavelength range, a pinhole setting of 1 AU (31 μm), 500 V detector gain, and 0 offset. Alexa Fluor 555: 535–617 nm emission wavelength range, a pinhole setting of 1.13 AU (31 μm), 600 V detector gain, and 0 offset. Multicolor widefield imaging (Figures 5 and 6) was performed on a Nikon Eclipse Ti2‐E inverted microscope fitted for a 25 mm field‐of‐view equipped with a Plan Apo λ 20× / NA 0.75 objective lens and a Photometrics Kinetix sCMOS camera. Illumination was provided by a CoolLED pE‐800 LED illumination system, using the 470 (Semrock FF01‐474/27–25, ~1.6 mW), 550 (Semrock FF01‐554/23–25, ~2.2 mW), and 635 (FF01‐635/18–25, ~1.8 mW) channels. Emission was separated from excitation by a Semrock FF409/493/573/652/759‐Di01‐25 × 36 polychroic mirror and further filtered using a Semrock FF01‐432/515/595/681/809‐25 pentaband emission filter. Images were recorded sequentially using 700 ms exposure time for each channel, a bit depth of 16 bits, no cropping, no binning, and no additional magnification, resulting in 320 × 320 nm pixels and a total image size of 1033 × 1033 μm. All images were analyzed with Fiji ImageJ open‐source software. All analyses were conducted by observers blinded to the mouse genotypes. Used antibodies are summarized in Table S2.

### 2.9 Statistical Analysis

All statistical testing was performed using GraphPad Prism 10.0.3.275. (San Diego, CA, USA). Two‐tailed Mann–Whitney *U*‐tests were used to compare the genotypes. Data are reported as mean ± standard error of the mean (SEM). The numbers of animals used for experimental comparison are specified in the figure legends. Significant outliers were identified and excluded from the data by using the ROUT method with a *q*‐value of 1%. A *p*‐value smaller than 0.05 was considered statistically significant. In the figures, asterisks denote statistical significance: *p < 0.05, **p < 0.01, and ***p < 0.001.

## 3 Results

### 3.1 Elevated *APP* Expression and Intraneuronal Amyloid‐β in the Myenteric Plexus of 5xFAD Mice

**FIGURE 1:**
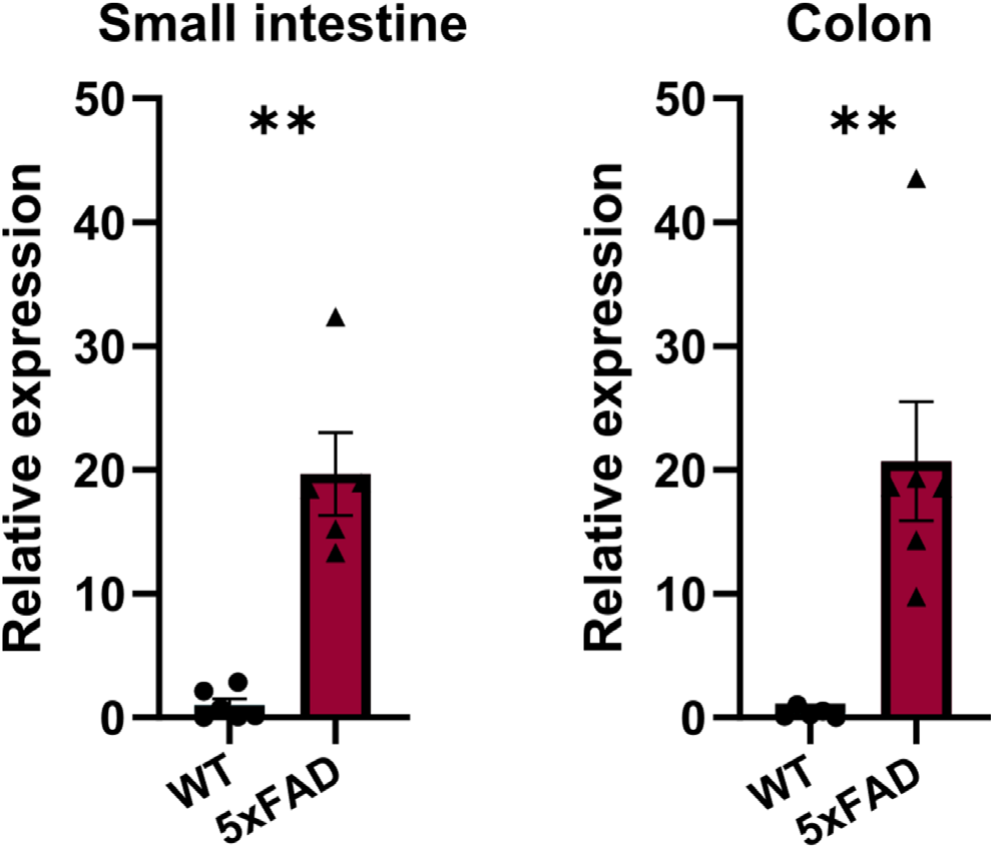
*APP* expression in small intestinal (left) and colonic (right) myenteric plexus preparations isolated from WT and 5xFAD mice as determined by RT‐qPCR. Increased expression of *APP* was detected in both regions in 5xFAD as compared to WT mice (*N* = 5 per genotype, **p < 0.01).

**FIGURE 2:**
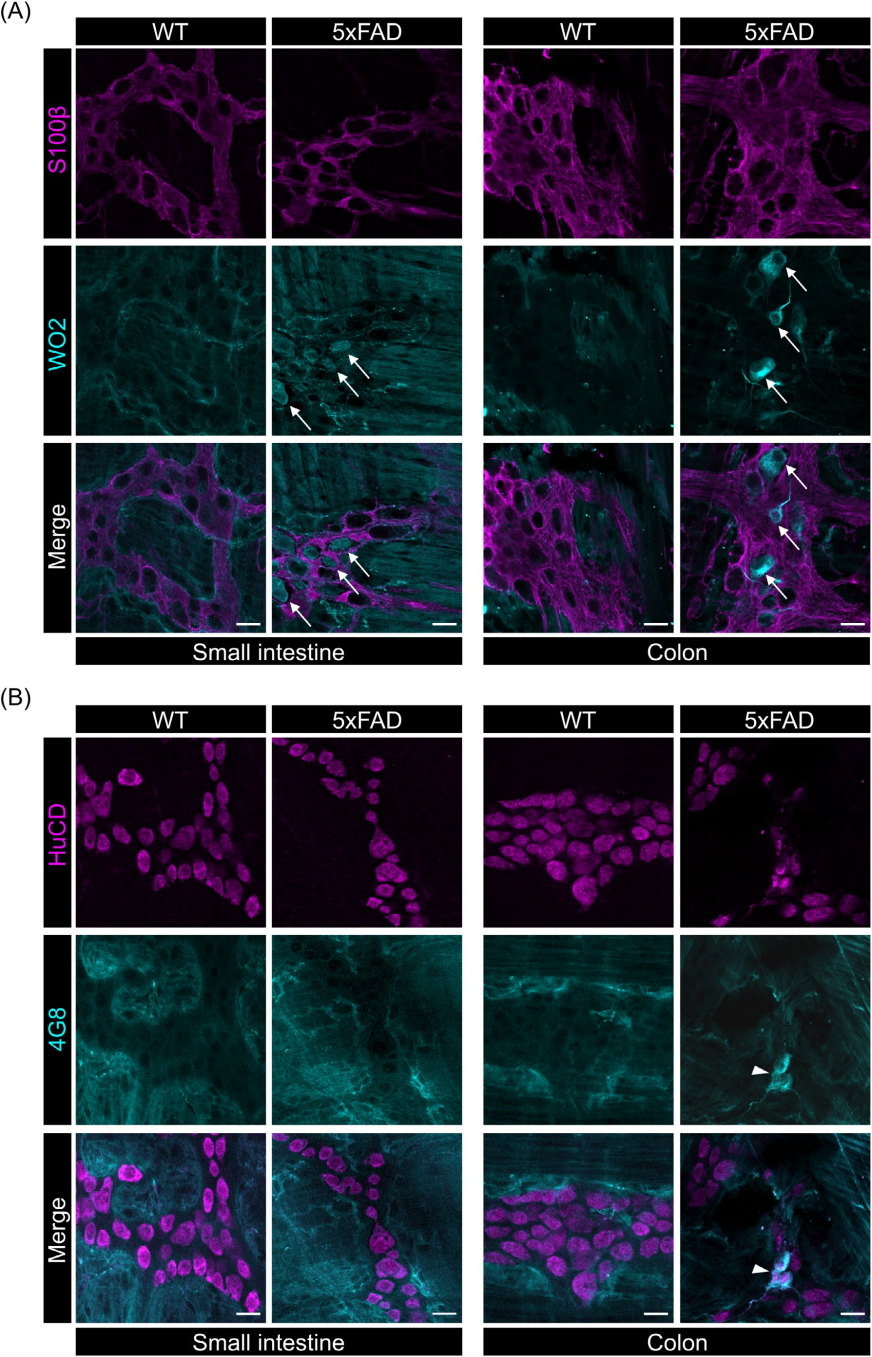
Aβ presence in the myenteric plexus. (A) Representative confocal images (*N* = 4 per genotype) of small intestinal and colonic myenteric plexus preparations immunolabeled for S100ß and WO2, showing the enteric glia network (magenta) and Aβ (turquoise), respectively. Aβ accumulation can be found in colonic myenteric ganglia (arrows). (B) Representative (*N* = 4 per genotype) confocal images of small intestinal and colonic myenteric plexus preparations immunolabeled for HuCD and 4G8, showing enteric neurons (magenta) and Aβ (turquoise), respectively. Intracellular accumulation of Aβ was detected in myenteric neurons in the colon of 5xFAD mice (arrowheads). Scale bars: 20 μm.

**FIGURE 3:**
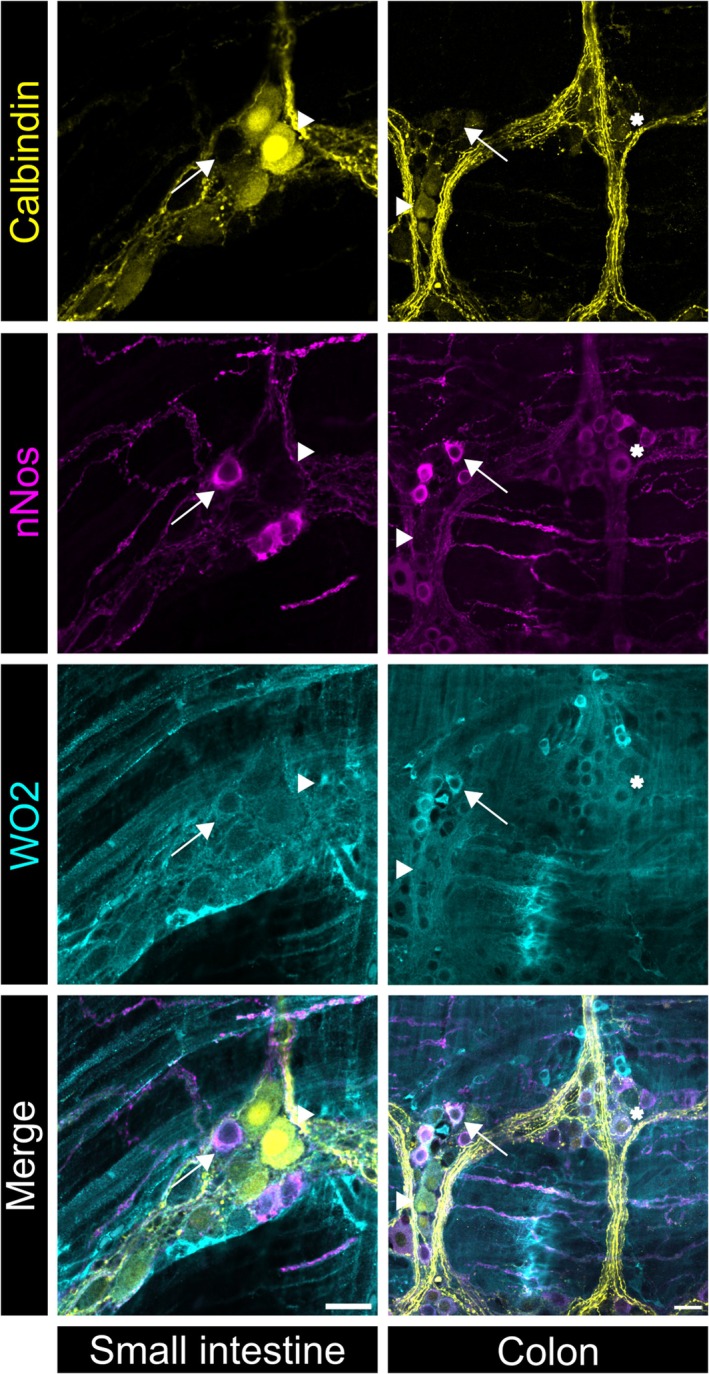
Aβ‐positive myenteric neurons in the small intestine and colon of 5xFAD mice. Representative confocal images (*N* = 4 per genotype) of myenteric neurons in the small intestine and colon immunolabeled for WO2 (turquoise) and the enteric neuron subtype markers calbindin (yellow) and nNOS (magenta). Intracellular accumulation of Aβ was detected both in calbindin (arrowheads) and nNOS (arrows) expressing myenteric neurons. The asterisk depicts a myenteric neuron that is positive for WO2, calbindin, and nNOS. Scale bars: 20 μm.

To investigate the presence of APP in the gut of 5xFAD mice, qPCR analysis was conducted on myenteric plexus preparations obtained from 6‐month‐old animals. Significantly elevated levels of *APP* gene expression were found in myenteric plexus preparations of the small intestine and colon when compared to WT littermates (Figure 1). Although no extracellular Aβ plaques or deposits were detected in the gut of 5xFAD mice (data not displayed), immunofluorescence labeling using the anti‐Aβ‐directed antibodies WO2 and 4G8 revealed intracellular Aβ accumulation in myenteric neurons in both the small intestine and colon of 5xFAD mice (Figure 2A,B). By combining WO2 staining with labeling for calbindin or neuronal nitric oxide synthase (nNOS), we did not find intracellular Aβ accumulation to overlap predominantly with either one of the enteric neuron subtype markers (Figure 3).

### 3.2 Low‐Grade Inflammation in the Gastrointestinal Tract of 5xFAD Mice

**FIGURE 4:**
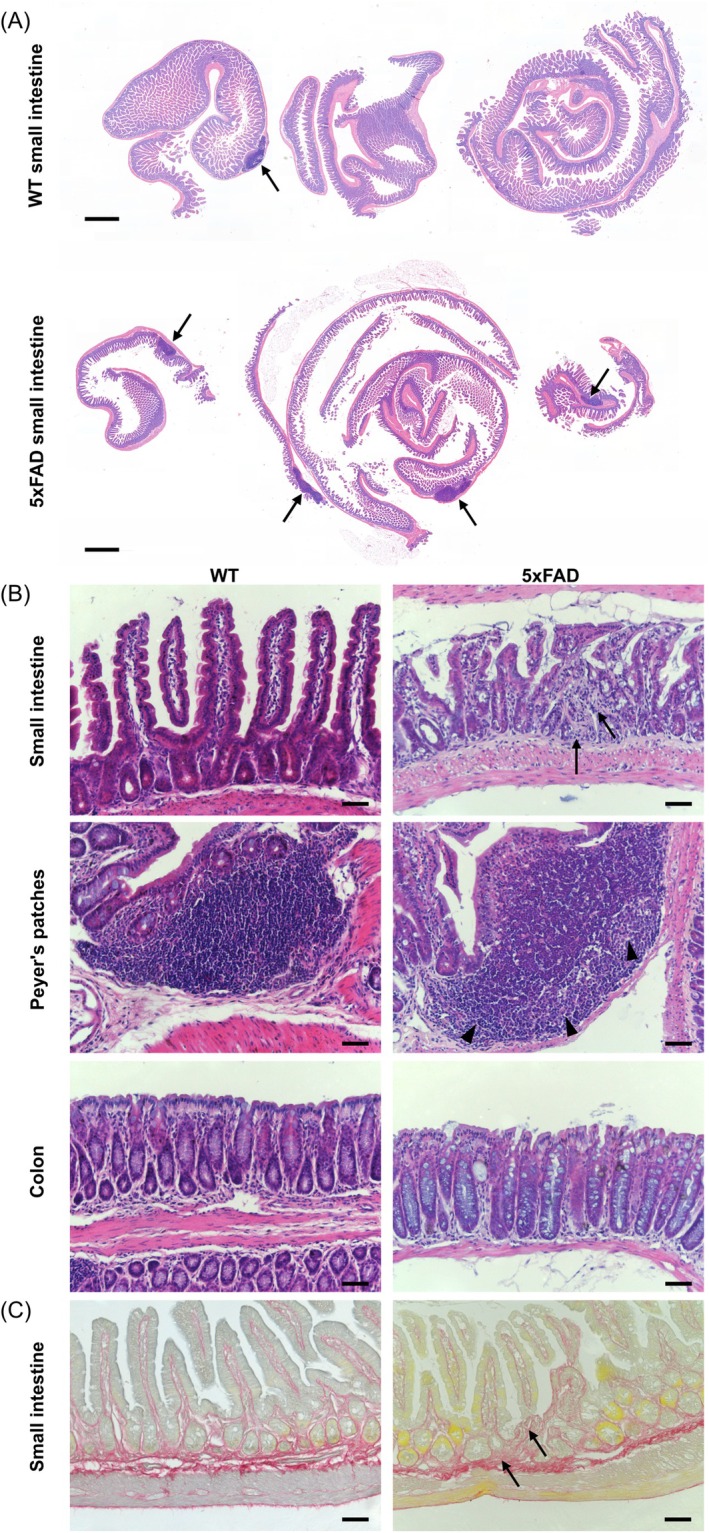
Enlarged Peyer's patches and fibrosis in the gastrointestinal tract of 5xFAD mice. (A) Low‐power microscopic images of H&E‐stained sections (*N* = 6 per genotype) showing an increased number of Peyer's patches (arrows) in the small intestine of 5xFAD mice. Scale bars: 1 mm. (B) Zoomed‐in H&E‐stained sections of Swiss rolls (*N* = 6 per genotype) illustrate pathological changes in the intestinal wall of 5xFAD mice. Arrows in the top panels indicate scar tissue accumulation in the small intestine. Middle panels show enlarged and activated Peyer's patches with prominent germinal centers (indicated by arrowheads) distributed along the gut in 5xFAD mice. No abnormalities were observed in the colon. Scale bars: 50 μm. (C) Representative images of Sirius red staining of the small intestine. Arrows indicate regions of fibrotic tissue. Scale bars: 50 μm.

**FIGURE 5:**
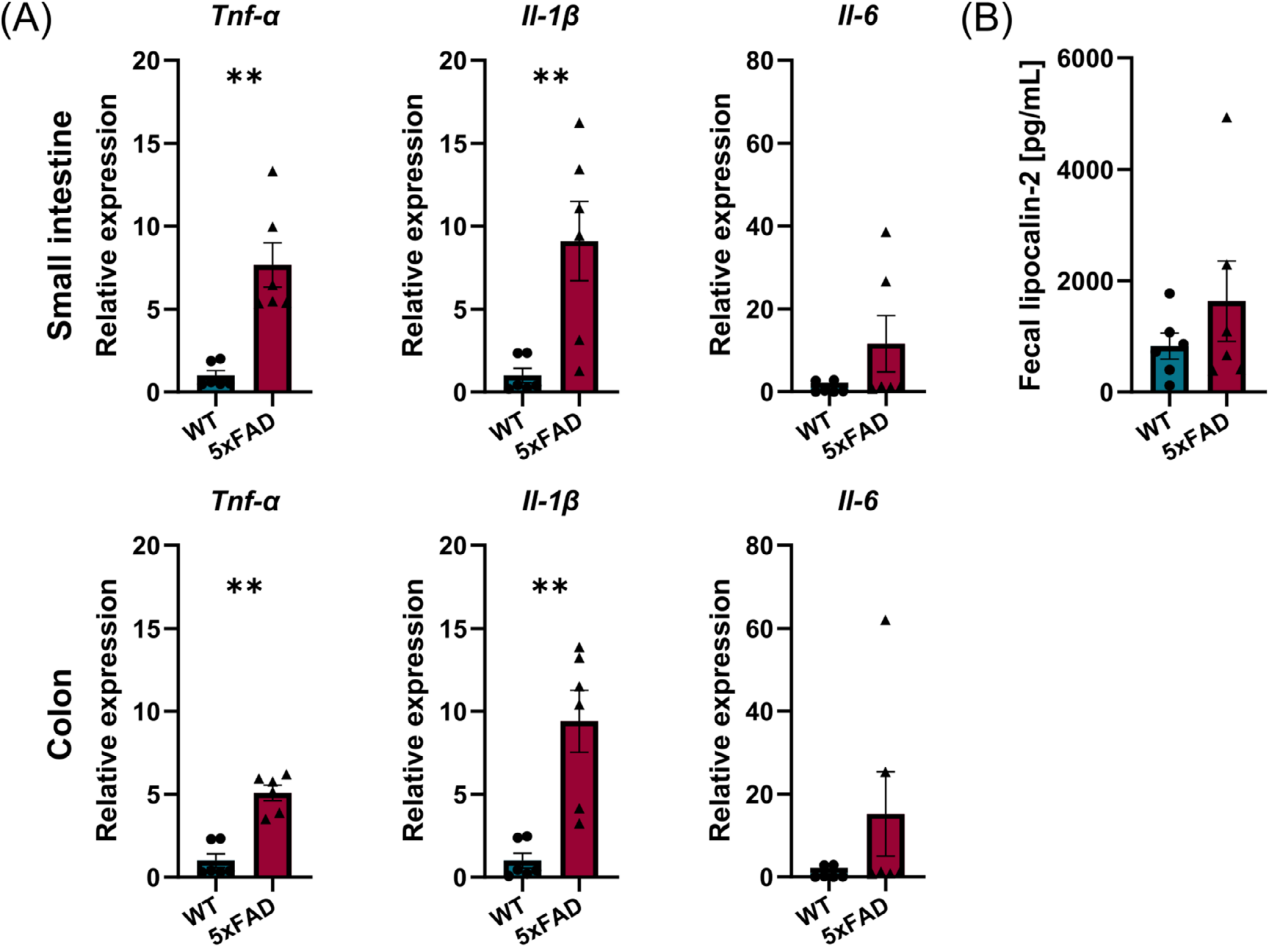
Expression of proinflammatory cytokines in 5xFAD mice. (A) Elevated levels of proinflammatory cytokine mRNAs were found in whole gut tissue samples from both the small intestine and colon by RT‐qPCR (*N* = 6 per genotype, **p < 0.01). (B) Fecal lipocalin‐2 levels were not significantly increased in 5xFAD mice (*N* = 6 per genotype).

To evaluate intestinal tissue architecture and potential histopathological alterations in 5xFAD mice, H&E staining was performed on small intestinal and colon Swiss‐rolls. In 5xFAD small intestines, we observed mild inflammation and intestinal fibrosis, characterized by an accumulation of inflammatory cells and by the presence of scar tissue in the intestinal wall (Figure 4), which was also evident from Sirius red staining (Figure 4C). Additionally, enlarged and activated Peyer's patches were found throughout the gut in 5xFAD mice, both in the small intestine and colon (Figure 4A,B). Of note, other organs (lung, liver, kidney, and spleen) appeared normal (data not shown). The presence of low‐grade intestinal inflammation was further confirmed by an increase in mRNA levels of intestinal inflammatory cytokines, namely *Tnf‐α, Il‐1β*, and *Il‐6*, which were analyzed in whole gut tissue samples from the small intestine and colon. Significant increases in *Tnf‐α* and *Il‐1β* levels were observed, while *Il‐6* showed a small, albeit non‐significant, increase in 5xFAD mice in both small intestine and colon samples (Figure 5A). However, assessment of lipocalin‐2 levels in stool samples, as another measure for intestinal inflammation, revealed no significant difference between the two genotypes (Figure 5B). Together, these data indicate that 5xFAD mice display mild intestinal inflammation and fibrosis.

### 3.3 Altered Gastrointestinal Motility in 5xFAD Mice

**FIGURE 6:**
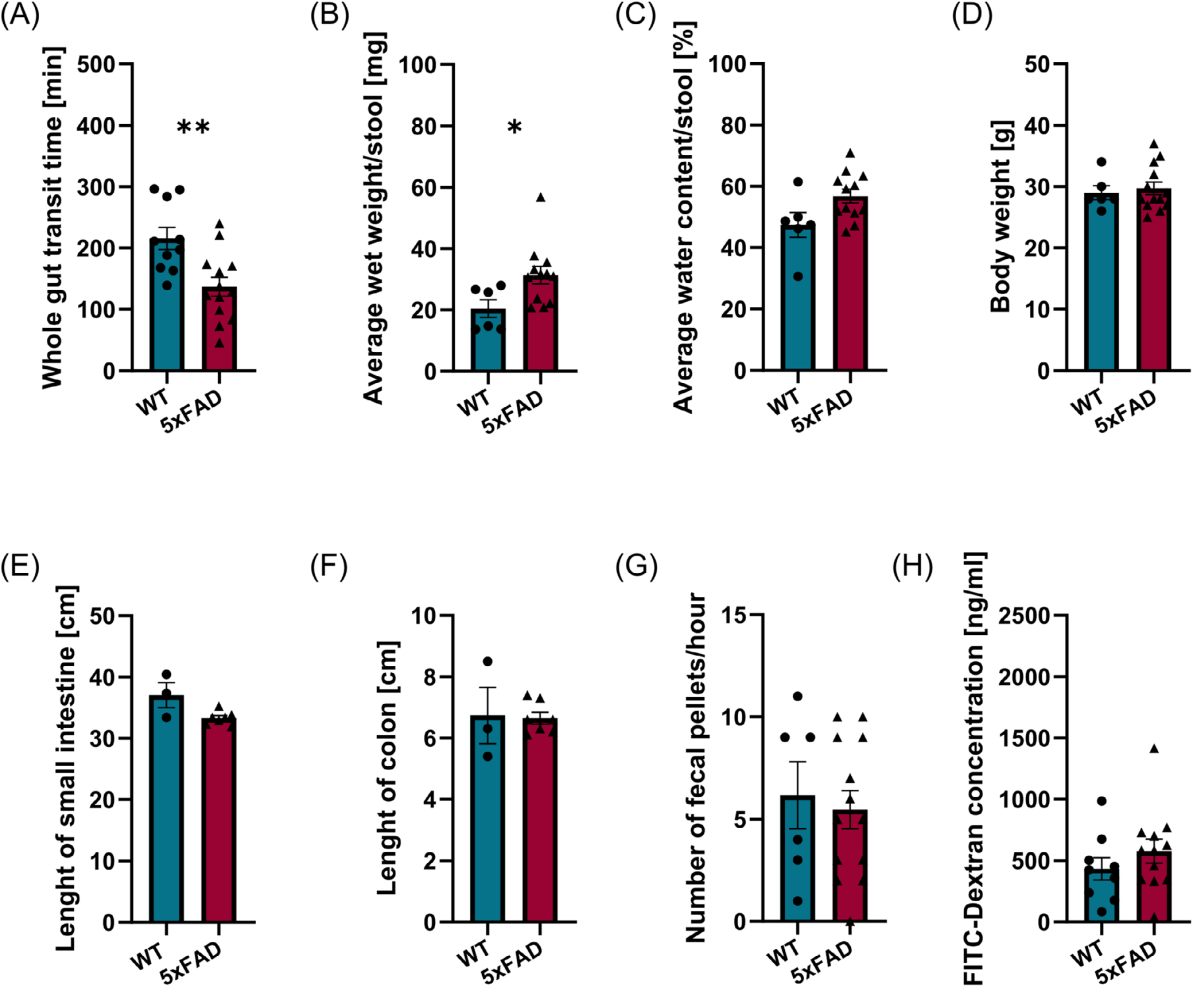
Gastrointestinal dysfunction in 5xFAD mice. (A) Whole gut transit is faster in 5xFAD mice relative to their WT littermates (*N* = 10–13 per genotype). This is accompanied by (B) an increase in the average wet weight (*N* = 6–13 per genotype) and (C) water content of stools (*N* = 6–13 per genotype). No differences were observed between 5xFAD and WT littermates regarding (D) mouse weight (*N* = 6–13 per genotype), (E, F) gut length (*N* = 3–7 per genotype), and (G) defecation frequency (*N* = 6–13 per genotype). (H) intestinal permeability is not affected in 5xFAD mice (*N* = 9–12 per genotype). *p < 0.05, **p < 0.01.

Whole gut transit time measurements revealed that 6‐month‐old 5xFAD mice exhibited faster gastrointestinal transit as compared to WT littermates (Figure 6A). This was accompanied by an increase in the average wet weight and water content of stools (Figure 6B,C). No differences were observed between 5xFAD and WT littermates in terms of the weight of the mice, gut length, and frequency of defecation (Figure 6D–G). Furthermore, intestinal permeability, as measured by the 4 kDa FITC‐Dextran barrier function assay, did not differ between 5xFAD mice and their WT littermates (Figure 6H).

### 3.4 ENS Composition Is Unaltered in 5xFAD Mice

**FIGURE 7:**
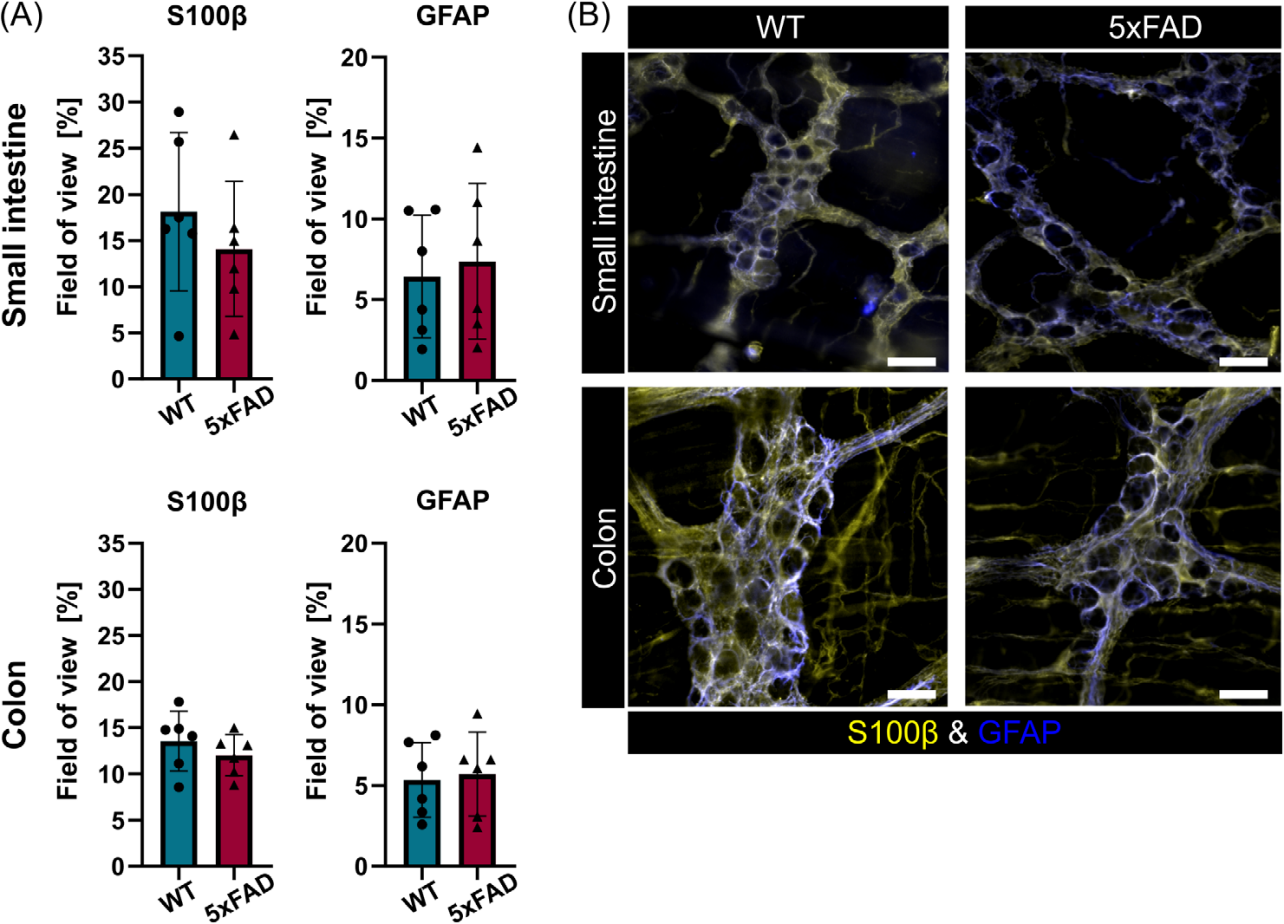
Enteric glial cells are not affected in 5xFAD mice. (A) Analysis of enteric glial network organization revealed no differences between the two genotypes (*N* = 6 per genotype). (B) Representative immunofluorescence images of small intestinal and colonic myenteric plexus preparations labeled for S100B (yellow) and GFAP (blue) (*N* = 6 per genotype). Scale bars: 20 μm.

**FIGURE 8:**
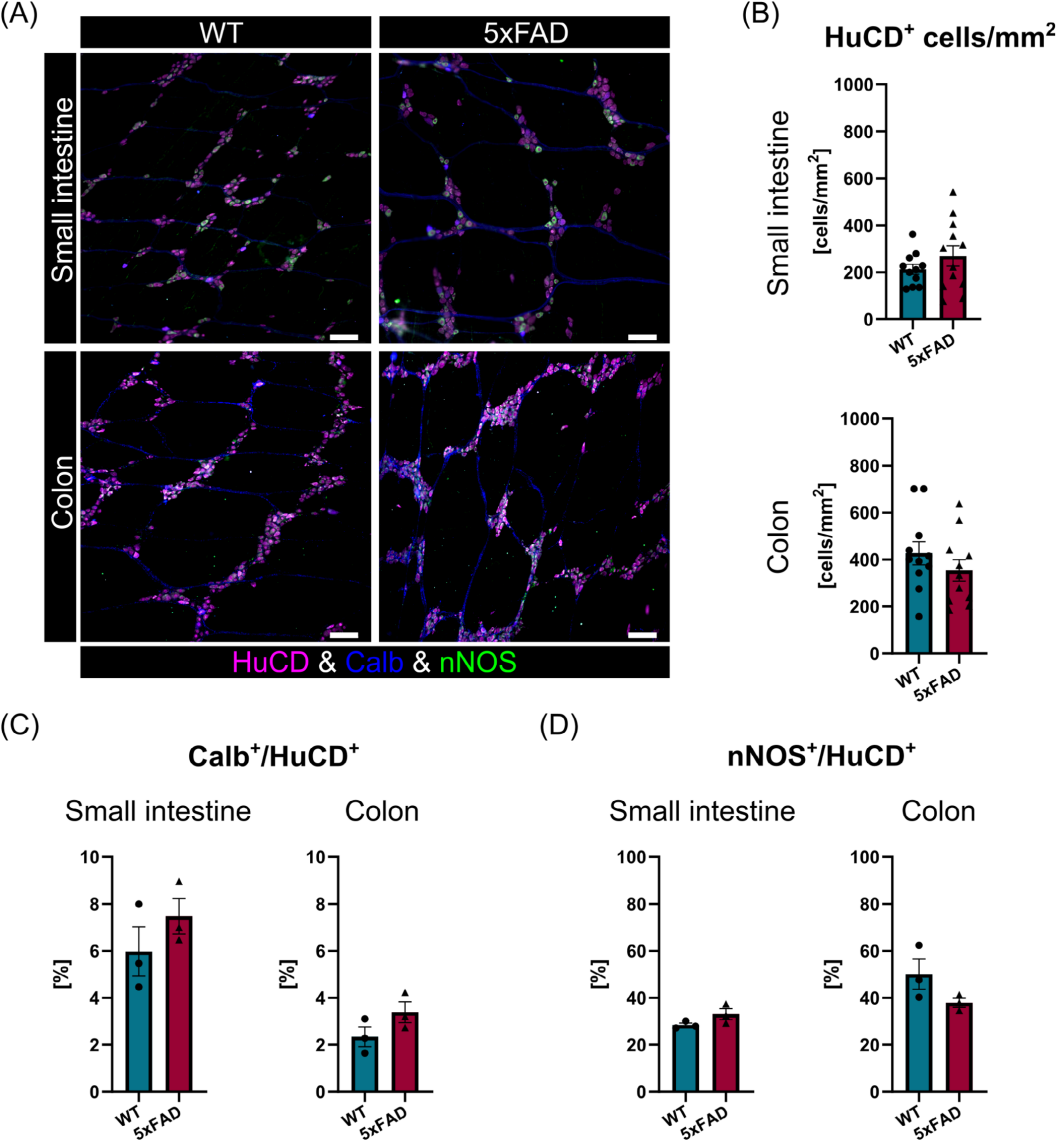
Enteric neuron numbers and specific subtype composition are not affected in 5xFAD mice. (A) Representative immunofluorescence images showing HuCD (magenta), calbindin (blue), and nNOS (green)‐positive myenteric neurons in the small intestine and colon. Scale bars: 100 μm. (B) Comparison of the number of myenteric neurons (HuCD, *N* = 11 per genotype) and (C, D) proportion of Calbindin (*N* = 3 per genotype) and nNOS (*N* = 3 per genotype) positive myenteric neurons in 5xFAD mice and WT littermates.

Quantification of enteric glial network organization revealed no differences between the two genotypes (Figure 7A,B), and immunofluorescence labeling for HuCD demonstrated that 5xFAD mice had a similar myenteric neuron count compared to WT littermates (Figure 8A,B). In addition, interganglionic distances in the small intestine (WT: 140.2 ± 9.152 μm vs. 5xFAD: 126.3 ± 9.817 μm, *p* = 0.400) or colon (WT: 158.0 ± 20.760 μm vs. 5xFAD: 171.8 ± 16.920 μm, *p* > 0.0999) did not differ significantly between the genotypes. Also, we did not observe changes in the number of calbindin or nNOS‐expressing neurons in 5xFAD mice (Figure 8C,D). Together, these data suggest that neuronal Aβ accumulation has no discernible effect on ENS composition.

## 4 Discussion

Neurodegenerative disorders are perceived as conditions primarily affecting the brain. However, recent studies increasingly acknowledge that peripheral processes contribute to disease onset and progression [34]. Also, emerging evidence has substantiated the association between AD pathology and gastrointestinal complaints [8]. We opted for the 5xFAD mouse model, a widely used preclinical AD model to investigate Aβ pathology and associated neuroinflammation [35, 36, 37], in an endeavor to elucidate the mechanisms behind AD‐related gastrointestinal dysfunction. Considering previous conflicting findings about ENS composition and gut function in studies employing this mouse model, our primary objective was to deliver a thorough pathological characterization of the 5xFAD model.

In a study conducted by Stoye et al., Aβ was identified using Western blotting in myenteric plexus preparations from adult 5xFAD mice; however, the exact cellular location remained unclear [24]. Our immunostainings using two different antibodies for W02 and 4G8 revealed the presence of Aβ accumulation within neurons of the myenteric plexus in 6‐month‐old 5xFAD mice. Although the *Thy1* promoter has mostly been used to drive transgene expression in the central nervous system, previous reports have demonstrated its activity in enteric neurons [38, 39, 40]. Whilst requiring local β‐ and ƴ‐secretase activity, the detection of APP products in the ENS of 5xFAD mice, therefore, is not surprising and is in agreement with earlier work showing potential Aβ deposits in the myenteric plexus [41]. Furthermore, despite the presence of a vast amount of extra‐ganglionic cells and non‐neural tissue as a potential source for APP mRNA, elevated levels in 5xFAD mice were confirmed by qPCR analysis. Of note, and contrary to our findings, Yelleswarapu et al., reported the absence of Aβ accumulation in the gut of 5xFAD mice, as measured by ELISA and immunostaining [25]. Methodological disparities between the studies likely account for these discrepancies.

Neuroinflammation is an important hallmark in the brain of AD patients [42], and is also observed in the 5xFAD model [27]. Accordingly, we observed an upregulation of proinflammatory cytokines in both the small intestine and colon of 6‐month‐old 5xFAD mice. Histological characterization revealed mild intestinal inflammation and fibrosis, which was associated with enlarged and activated Peyer's patches throughout the gut. These data indicate that mutant human *APP* and *PSEN1* transgenes driven by the *Thy1* promoter also cause inflammation in the periphery. Whether this is a direct consequence of Aβ accumulation in the ENS or secondary to alterations in intestinal microbiota in 5xFAD mice [41], is currently not clear. Indeed, it is well‐established that microbial dysbiosis can trigger an inflammatory response in the gastrointestinal tract [43, 44]. Also, an alternative scenario where the combination of dysbiosis and intestinal inflammation instructs the production and build‐up of abnormal protein accumulation in the gut, similar to AD pathology in the brain [45], might be plausible. It is noteworthy that inflammatory signals and microbial metabolites from the gut have been shown to traverse the gut‐brain axis, potentially contributing to AD‐related neuroinflammation in the CNS [16, 45]. Additionally, disrupted immune responses caused by dysbiosis may hinder the clearance of pathologically relevant proteins in the brain [46]. A similar phenomenon may contribute to the generation of Aβ accumulation in the gut of 5xFAD mice.

In keeping with previous observations [24, 25], our results indicate that 6‐month‐old 5xFAD mice exhibit accelerated gastrointestinal transit. However, our experiments show that ENS composition and organization generally appear normal in 5xFAD mice. Myenteric neuron counts and the number of calbindin and nNOS‐expressing neurons were unaffected, suggesting that enteric neuron loss is not present in 5xFAD mice at this stage, which is unlike findings in other AD mouse models [28, 29]. Next to enteric neurons, we also examined enteric glia networks in the 5xFAD mice. Enteric glial cells are very sensitive to changes in their microenvironment [47] and promptly adapt their phenotype in response to insults such as infection, ischemia, and intestinal surgery [48]. In this context, also bacterial amyloids have recently been shown to induce a proinflammatory state in enteric glia [49]. However, we did not detect changes in the enteric glia network, contradicting previous findings of increased colonic GFAP expression [24]. Together, these data demonstrate that gastrointestinal motility is not altered by dramatic changes in ENS structure. While gut function can be influenced by alterations in microbial composition, a phenomenon previously observed in this model [41], our findings suggest that gastrointestinal function is most likely affected by subtle defects in ENS circuitry and activity caused by Aβ accumulation and inflammation [50, 51]. Together with differences in intestinal microbial status, distinct levels of Aβ accumulation and the extent of inflammation probably also underlie the apparently irreconcilable motility phenotypes reported in 5xFAD mice [18, 24, 25, 26] and other APP/PS1 models with amyloid pathology [21, 22, 23].

In conclusion, our study sheds light on the complex interplay between AD pathology and gastrointestinal dysfunction. By characterizing peripheral pathology in the 5xFAD model, we detected Aβ accumulation within enteric neurons and showed that 5xFAD mice display mild intestinal inflammation. Aβ accumulation in the ENS and enteric neuroinflammation, together, likely contribute to AD‐related gut dysfunction. Our findings underscore the importance of peripheral pathology in AD.

## Author Contributions

K.R.T. and W.B. conceived and designed the experiments. K.R.T., R.J., and M.Gd.R. performed experiments and analyses. K.R.T. drafted the manuscript. M.J.G. performed histopathological analyses. Study supervision by V.M. and W.B. M.Gd.R., B.B., I.D., V.M., and W.B. contributed to editing and revising the manuscript. All authors approved the final manuscript.

## Conflicts of Interest

The authors declare no conflicts of interest.

## Supporting information


Data S1.
**Table S1**. List of primers used for RT‐qPCR. Gapdh was used as a housekeeping gene for the qPCR experiments testing the mRNA levels of *APP*, while *Pgk1* was used as a housekeeping gene for *Tnf‐α*, *Il‐1β*, and *Il‐6* mRNA measurements.
**Table S2**. List of antibodies used for immunofluorescence.

## Data Availability

The data that support the findings of this study are available from the corresponding author upon reasonable request.
