# Supplementary material for: Gastrointestinal Dysfunction and Low‐Grade Inflammation Associate With Enteric Neuronal Amyloid‐β in a Model for Amyloid Pathology
Source: Neurogastroenterol Motil. 2025 Mar 6;37(5):e15016. doi: 10.1111/nmo.15016 (PMC11996054; doi:10.1111/nmo.15016)
Supplement: Supplementary file 1 — Data S1. Table S1. List of primers used for RT‐qPCR. Gapdh was used as a housekeeping gene for the qPCR experiments testing the mRNA levels of APP, while Pgk1 was used as a housekeeping gene for Tnf‐α, Il‐1β, and Il‐6 mRNA measurements. Table S2. List of antibodies used for immunofluorescence. [file NMO-37-e15016-s001.pdf]

SUPPLEMENTARY TABLES

amyloid precursor protein (*APP*)	forward	CCATCAGGGACCAAAACCTGC
	reverse	TGGTCACTGGTTTGGCT
tumor necrosis factor alpha (*Tnf-α*)	forward	GCCACCACGCTCTTCTGTCTA
	reverse	ATGAGAGGGAGGCCATTTGGG
interleukin-1 beta (*Il-1β*)	forward	TGACCTGGGCTGTCCTGATG
	reverse	TCCGACAGCACGAGGCTTTT
interleukin-6 (*Il-6*)	forward	TGTTCTCTGGGAAATCGTGGA
	reverse	CTGCAAGTGCATCATCGTTGT
glyceraldehyde-3-phosphate dehydrogenase (*Gapdh*)	forward	TCCTGCACCACCAACTGCTT
	reverse	CACGCCACAGCTTTCCAGAG
phosphoglycerate kinase 1 (*Pgk1*)	forward	GAAGGGAAGGGAAAAGATGC
	reverse	GCTATGGGCTCGGTGTGC

Supplementary Table 1: List of primers used for RT-qPCR. *Gapdh* was used as a housekeeping gene for the qPCR experiments testing the mRNA levels of *APP*, while *Pgk1* was used as a housekeeping gene for *Tnf-α, Il-1β*, and *Il-6* mRNA measurements.

Primary antibody	Working dilution	Supplier of the primary antibody	Secondary antibody	Working dilution	Supplier of the secondary antibody
human α HuCD	1:20000	Kind gift from Dr. Vanda A. Lennon	goat α human	1:1000	Invitrogen (A-21433)
sheep α nNOS	1:3000	Kind gift from Dr. Piers Emson	donkey α sheep	1:1000	Invitrogen (A-11015)
rabbit α Calbindin	1:8000	Swant (CB-38a)	goat α rabbit	1:1000	Invitrogen (A-21245)
rabbit α S100β	1:400	Proteintech (15146-1-AP)	goat α rabbit	1:1000	Invitrogen (A-11008)
chicken α GFAP	1:1000	Abcam (ab4674)	goat α chicken	1:1000	Invitrogen (A-21449 & A-11039)
mouse α WO2 – clone	1:500	Merck/ Sigma-Aldrich (MABN10)	goat α mouse	1:1000	Invitrogen (A-28180)
mouse α 4G8 – clone	1:1000	BioLegend (Covance – SIG-39220)	goat α mouse	1:1000	Invitrogen (A-28180)

Supplementary Table 2: List antibodies used for immunofluorescence.
